# F190. EFFECT OF SELECTED GENE VARIANTS ON THE RELATIONSHIP BETWEEN EARLY CANNABIS USE AND AGE OF ONSET OF PSYCHOSIS

**DOI:** 10.1093/schbul/sby017.721

**Published:** 2018-04-01

**Authors:** Rohit Lodhi, Yabing Wang, Gina McIntyre, David Rossolatos, Sudhakar Sivapalan, Beatriz Henriques, Brodie Heywood, Candice Crocker, Scot Purdon, Kathy Aitchison, Philip Tibbo

**Affiliations:** 1 University of Alberta; 2 Dalhousie University, Nova Scotia Health Authority; 3 Capital District Health Authority

## Abstract

**Background:**

Cannabis use, particularly regular use in adolescence, is associated with an increased risk of developing psychosis earlier. An earlier age of onset negates the protective effects of more mature psychosocial and individual variables, thus the potential for worse outcomes. Genetic variations in this relationship are important to understand as this will allow not only a better understanding of the biological interaction of cannabis and psychosis, but would inform future genetic approaches to risk identification as well. We uniquely examined the mediation of this association (gene x cannabis associations in age of onset of psychosis (AoP)) in 3 genetic variants which, while each have been examined separately, not in combination in the same population. We examined: 1) COMT Val158Met (rs4680) 2) BDNF Val66Met (rs6265) and 3) the AKT1 variant rs2494732.

**Methods:**

168 subjects with a diagnosis of psychosis were recruited from 2 sites in Canada, Edmonton Alberta and Halifax Nova Scotia. Cannabis use data (age at first and regular use) were collected using an electronic self-report survey (to address potential minimization of use to a researcher) and saliva samples were used for genotyping. Kaplan-Meier and Cox regression analyses were used to study the gene – cannabis effects.

**Results:**

In those who had used cannabis, first use of cannabis prior to 20 years of age was associated with earlier AoP (p = .005). In those who used cannabis before age 20, rs4680 had a trend level association with AoP (log rank test: p=0.0617). A trend effect for an rs6265 x gender interaction (HR = 2.08, p = 0.067) on AoP, controlling for regular cannabis use was also observed. No association was observed between rs2494732 and rs6265 - rs2494732 interaction, and AoP.

**Discussion:**

The trends in our associations are in keeping with previous literature, however our gender analyses underscores the importance of examining sex and gender as we further move towards risk identification for the cannabis and psychosis interaction. Our results also suggest that not all genetic variants associated with psychosis are involved with the association between cannabis and AoP. While our results offer support for future research in this area, larger sample sizes are required to test the gene-cannabis-AoP relationship.

